# Comparison of decomposition of biodegradable mulching films under variable soil microbial conditions

**DOI:** 10.3389/fmicb.2025.1674576

**Published:** 2025-09-24

**Authors:** Sung Jae Kim, Jun-Yeop Shim, Kyoung Min Park, Dong il Park, Na Young Heo, Su Jin Hwang, Sung Hoon Park, Hyeon Woo Chung, Jae Myun Lee, Hee Chun Chung

**Affiliations:** ^1^Department of Companion Animal Health, Kyungbok University, Namyangju, Republic of Korea; ^2^R&F Chemical Co., Ltd., Hanam-si, Republic of Korea; ^3^Department of Microbiology and Immunology, Institute for Immunology and Immunological Diseases, Yonsei University College of Medicine, Seoul, Republic of Korea; ^4^Department of Microbiology and Immunology, Institute for Immunology and Immunological Diseases, Brain Korea 21 Project for Medical Science, Yonsei University College of Medicine, Seoul, Republic of Korea

**Keywords:** biodegradable mulching films, *Bacillus subtilis*, *Clostridium perfringens*, pH, soil

## Abstract

**Introduction:**

Biodegradable mulching films (BDMs) are sustainable alternatives to polyethylene, but their degradation efficiency is strongly influenced by soil microbial composition. This study investigated the effects of *Bacillus subtilis* and *Clostridium perfringens*, two soil bacteria with distinct metabolic traits, on the decomposition of BDMs with different structures.

**Methods:**

Three biodegradable films (BDM1, BDM2, BDM3) and a polyethylene control were buried in soils containing native microbes, *B. subtilis*, or *C. perfringens* and incubated for 210 days. Degradation was evaluated by weight loss, soil pH, microbial viability, and scanning electron microscopy (SEM) of surface morphology.

**Results:**

All BDMs degraded significantly more than polyethylene. The monolayer BDM3 exhibited the greatest weight loss and surface damage. Soils inoculated with *C. perfringens* underwent strong acidification (final pH < 5.5), which accelerated degradation, especially in CaCO₃-containing films. Although *C. perfringens* viability declined over time, accumulated acids sustained film breakdown. By contrast, *B. subtilis* maintained higher soil populations, promoted gradual degradation, and preserved near-neutral pH, resulting in moderate weight loss.

**Discussion:**

These findings demonstrate that soil pH modulation and microbial activity jointly determine BDM degradation. While *C. perfringens* enhanced film loss through acidification, its agricultural use may pose risks including excessive soil acidification and pathogenicity. *B. subtilis* provided safer but slower biodegradation. Among the tested films, monolayer BDM3 was most susceptible to breakdown, making it a promising candidate for field application. Careful management of microbial inoculants and soil pH will be essential to maximize BDM performance and environmental safety.

## 1 Introduction

The increasing global demand for plastic-based agricultural inputs, particularly polyethylene mulch films, has raised critical concerns regarding their long-term environmental persistence and ecological consequences (Sintim and Flury, 2017). Conventional plastic mulching films (PMFs) are widely used to suppress weeds, retain soil moisture, and enhance crop productivity (Lamont, 2017). However, these benefits are offset by the recalcitrant nature of petroleum-based polymers, which accumulate in soils as microplastics and interfere with soil structure, microbial ecology, and long-term fertility (Qi et al., 2018; Sintim et al., 2020). To address these limitations, biodegradable mulching films (BDMs) have been developed using biodegradable polymers such as polylactic acid (PLA), polybutylene adipate terephthalate (PBAT), and starch blends (Kasirajan and Ngouajio, 2012). These materials are engineered to break down through microbial activity under natural conditions, thereby minimizing long-term environmental burden (Bandopadhyay et al., 2023). Despite their promise, the biodegradation efficiency of BDMs is not uniform and is strongly influenced by edaphic factors, temperature, moisture, and most critically, the composition and activity of soil microbial communities (Bandopadhyay et al., 2020a; Xue et al., 2022). Soil microorganisms are central to the transformation and mineralization of organic substrates, including synthetic polymers. Specific bacterial groups such as Bacillus, Pseudomonas, Streptomyces, and various Actinobacteria are known to produce extracellular enzymes (e.g., lipases, esterases, proteases) that catalyze the hydrolysis of polymer bonds (Lucas et al., 2008; Shah et al., 2008). Among these, *Bacillus subtilis*, a Gram-positive, spore-forming bacterium, is particularly notable for its high metabolic versatility, environmental stability, and enzymatic capacity. Previous studies have demonstrated its potential in the biodegradation of polymer composites and its use in bioremediation strategies (Setlow, 2016; Baskaran and Sathiavelu, 2022). Contrastingly, *Clostridium perfringens* typically regarded as a nosocomial pathogen, is also present in natural environments including compost, animal waste, and even agricultural soil (Liu et al., 2010; Chisholm et al., 2023). As a strict anaerobe and prolific spore-former, *Clostridium perfringens* is capable of surviving under oxygen-deprived and nutrient-depleted conditions (Liu et al., 2010). Its metabolic byproducts, particularly volatile fatty acids and ammonia, can significantly alter the pH and redox conditions of soil microenvironments, potentially influencing polymer degradation dynamics (Khoruts et al., 2021; Schnizlein and Young, 2022). Despite the known presence of these organisms in soil, relatively few studies have systematically compared the degradation behavior of BDMs under soils inoculated with specific, well-characterized bacterial strains (Bandopadhyay et al., 2020a; Bandopadhyay et al., 2020b).

In this study, we focused on the influence of two representative soil bacteria: *Bacillus (B.) subtilis*, a common facultative anaerobic decomposer, and *Clostridium (C.) perfringens*, a strict anaerobic spore-forming bacterium found in compost and soil environments. Through an experimental comparison of BDMs with varying polymer compositions and the evaluation of their degradation in soils inoculated with defined bacteria, this study underscores the potential of microbiome-based approaches to enhance the biodegradation of agricultural plastics.

## 2 Materials and methods

### 2.1 Biodegradable mulching film design and composition

All film types used in this study were developed by RNF Chemical Co., Ltd. Four types of films were prepared, including three biodegradable multilayer/mono-layer films (BDM1, BDM2, BDM3) and one non-biodegradable control film (NC), with detailed compositions as follows:

Four types of mulching films were used in this study, all of which were independently developed and manufactured by RNF Chemical Co., Ltd. These included three biodegradable films (BDM1, BDM2, BDM3) and one non-biodegradable control film (NC). Each film was formulated with distinct polymer blends and structural configurations to assess their relative degradation behaviors under varying soil microbial conditions. BDM1 was designed as a multilayer biodegradable film with a total thickness of 45 μm. It consisted of three layers: the two outer layers (15 μm each) were composed of a mixture of polybutylene adipate terephthalate (PBAT, 60%), calcium carbonate (CaCO₃, 30%), and polylactic acid (PLA, 10%), while the middle layer (15 μm) contained PBAT (100%) to enhance mechanical strength and elasticity. BDM2 was also fabricated as a three-layer biodegradable film with an identical total thickness of 45 μm (out layers: 15 μm each, middle layer: 15 μm). The outer layers comprised PBAT (50%), thermoplastic starch (TPS, 20%), and PLA (30%), whereas the middle layer included a 1:1 blend of PBAT (50%) and TPS (50%). This composition was selected to promote enhanced microbial accessibility and accelerate biodegradation through starch incorporation. BDM3 was prepared as a single-layer biodegradable film with a thickness of 45 μm, composed of PBAT (60%), CaCO₃ (30%), and PLA (10%). This simpler, homogenous structure allowed for direct comparison with the multilayer films in terms of biodegradability and microbial response. NC, the non-biodegradable control film, was produced as a single-layer film (45 μm) composed entirely of low-density polyethylene (LDPE, 100%). It served as a negative control to validate the selective degradation behavior observed in biodegradable film treatments.

All films were manufactured using a cast film extrusion process under standardized conditions.

Each film type was cut into two uniform rectangular samples (20 cm × 15 cm), and a total of two pieces per film type were used in the soil incubation. The final products were cut into uniform rectangular sections, and their thickness was verified using a calibrated micrometer. Film composition and layer structure were confirmed based on proprietary formulation records and material specification data provided by the manufacturer.

### 2.2 Soil preparation

The baseline soil used in this study, referred to as the original group, was a commercially available soil product (Taeheung F&G, South Korea), designed for use in the cultivation of vegetables, root crops, and ornamental plants. This soil was used as the source of the native microbial community without any modification. In addition to the soil with the native microbial community (Original), two artificial soil conditions were prepared using soil-abundant bacterial species (*B. subtilis* and *C. perfringens*) that may participate in the degradation of mulching films under agricultural conditions. The *B. subtilis* and *C. perfringens* strains used in this study were isolated from the original soil, respectively. Both isolates (*B. subtilis* and *C. perfringens*) was confirmed using matrix-assisted laser desorption ionization–time of flight mass spectrometry (MALDI-TOF MS; Microflex LT, Bruker Daltonics, Germany). Spectral profiles were analyzed using the Bruker Biotyper software, and identification scores were interpreted according to the manufacturer’s criteria: scores ≥ 2.0 were accepted for species-level identification. To create sterilized soil conditions for controlled microbial inoculation, a portion of the original soil was autoclaved at 121 °C for 20 min on two consecutive days to eliminate indigenous microorganisms and cooled to room temperature. Each bacterial strain was cultured under optimal growth conditions, harvested at late exponential phase, and introduced into the sterile soil at a final concentration of 5 × 10^6^ colony-forming units (CFU) per gram of soil. The inocula were thoroughly mixed with the soil using sterile spatulas to ensure even microbial distribution. Five kilogram of each prepared soil type was transferred into sterile polypropylene containers (60 cm × 40 cm × 30 cm, L × W × H) with perforated lids to allow limited gas exchange while maintaining humidity and appropriate redox conditions for microbial activity.

### 2.3 Film burial and incubation conditions

In each container, two rectangular film samples (20 cm × 15 cm) of a given film type were buried horizontally at a depth of 5 cm from the soil surface. The films were placed flat and parallel to the base of the container, spaced apart to avoid overlapping. The soil surface was then gently leveled to maintain consistency across all experimental replicates. All soil-film assemblies were incubated for a total of 210 days at a constant temperature of 25 ± 1 °C in a controlled-environment chamber. Soil moisture was maintained at 60% of water-holding capacity by weighing the containers and supplementing with sterile distilled water twice weekly to offset evaporation and microbial consumption.

These three soil treatments—Original (native microbial community), Bacillus-inoculated, and Clostridium-inoculated—were used to evaluate the degradation behavior of different mulching films under standardized and biologically defined soil microbial conditions over an extended incubation period.

### 2.4 Soil pH and film weight measurements

To monitor soil biochemical changes and film degradation dynamics, soil pH and film weight measurements were conducted simultaneously every 2 weeks for the duration of the 210-day incubation period, across all experimental groups (Original, Bacillus, and Clostridium treatments). For soil pH analysis, approximately 10 g of soil was collected from the upper 5 cm layer of each container near the location of buried films. The collected soil samples were air-dried and sieved (2 mm mesh) before analysis. Soil pH was measured using the pH METER DELTA 350 (Mettler Toledo, Switzerland), calibrated with standard buffer solutions (pH 4.01, 7.00, and 10.01) prior to each measurement session. The pH was determined in a 1:5 (w/v) soil-to-distilled water suspension, stirred for 30 min and allowed to equilibrate for 10 min before reading. For film weight loss assessment, one of the two buried film samples in each container was carefully retrieved at each time point (every 14 days) using sterile forceps. The film was gently rinsed with sterile distilled water to remove attached soil particles and then air-dried at room temperature for 24 h. Afterward, the samples were dried in a convection oven at 60 °C for 12 h to ensure constant weight. The final dry weight was recorded using an analytical balance (± 0.1 mg precision). Weight retention (%) was calculated relative to the initial pre-burial weight of each film sample. All measurements were performed in triplicate for each treatment group to ensure statistical reliability. The results were used to track temporal changes in both soil chemical properties and the extent of film biodegradation under different microbial conditions.

### 2.5 Quantification of bacterial viability in soil

To evaluate the persistence and viability of the inoculated bacterial strains over time, colony-forming unit (CFU) counts were conducted at monthly intervals throughout the 210-day incubation period for each treatment groups: Bacillus and Clostridium. At each time point (every 30 days), approximately 5 g of soil was aseptically collected from each container using sterile spatulas. Sampling was performed in the vicinity of the buried film samples to accurately represent the microbial environment directly interacting with the films. The soil samples were transferred into 50 mL conical tubes containing 45 mL of sterile phosphate-buffered saline (PBS, pH 7.2), then vortexed and shaken at 150 rpm for 30 min at room temperature to release viable bacteria into suspension. The resulting soil suspensions were serially diluted in 10-fold steps using sterile PBS. From each dilution, 100 μL aliquots were plated in triplicate onto selective agar media as follows: For the Bacillus group, samples were plated on nutrient agar (NA) and incubated aerobically at 37 °C for 24–48 h. For the Clostridium group, samples were plated on reinforced clostridial agar (RCA) and incubated anaerobically at 37 °C for 48 h using a GasPak anaerobic chamber (BD Diagnostics). After incubation, representative colonies on the plates were selected and were validated by MALDI-TOF. Final CFU counts were calculated and expressed as log₁₀ CFU per gram of dry soil. All procedures were performed in triplicate for each treatment group at each time point. These data were used to monitor the temporal dynamics of bacterial viability and to assess their potential role in facilitating the biodegradation of mulching films under different microbial soil conditions.

### 2.6 Surface morphology analysis by field emission scanning electron microscopy

To assess surface degradation features after long-term soil exposure, scanning electron microscopy (SEM) analysis was performed on all four film types (BDM1, BDM2, BDM3, and NC) retrieved from the Clostridium-inoculated soil after 210 days of incubation. This soil condition exhibited the highest overall weight loss among all microbial conditions.

After collection, film samples were gently rinsed with sterile distilled water to remove residual soil particles and air-dried at room temperature. The samples were then mounted on aluminum stubs using carbon tape and coated with a thin layer of platinum using an F-Pt-6-200 coating process to enhance conductivity. Surface morphology was observed using a field emission scanning electron microscope (FE-SEM; SU5000, Hitachi, Japan) operated at an accelerating voltage of 10 kV and beam intensity setting of 30. Micrographs captured from each sample at each of the three magnifications (×3,000, ×10,000, and ×30,000). For quantitative surface analysis, the images were selected for further image analysis using ImageJ software version 1.54 g (NIH, United States). For the quantitative analysis of surface roughness and degradation features using ImageJ, high-resolution SEM images were first converted to 8-bit grayscale. Thresholding was applied to distinguish surface features such as cracks, pores, and erosion pits. Particle analysis was then performed using the “Analyze Particles” function, with size and circularity parameters adjusted to exclude background noise and artifacts. Surface degradation was quantified based on the number, area, and distribution of the detected features. Additionally, the roughness index was calculated by analyzing the grayscale intensity variation across defined line profiles using the “Plot Profile” and “Measure” tools. All measurements were repeated in triplicate per image and averaged for statistical comparison between film type.

### 2.7 Statistical analysis

All quantitative data including soil pH, film weight retention, CFU counts, and SEM-based surface damage ratios were statistically analyzed in R v4.4.1 and results were visualized in R studio. The data for soil pH, film weight retention, and CFU counts were analyzed using linear mixed models (random effect: sample, fixed effects: group and time) with the lme4 package. *Post-hoc* pairwise comparisons between groups were performed using Tukey’s HSD test, which applies multiple comparison correction to control family-wise error rate across all pairwise tests. The data for SEM-based surface damage ratios were analyzed using the Kruskal–Wallis test, and pairwise comparisons between groups were subjected to multiple comparison adjustment using the Bonferroni correction method to control type I error inflation.

## 3 Results

### 3.1 Time-dependent changes in soil pH across treatment groups

**Figure 1:**
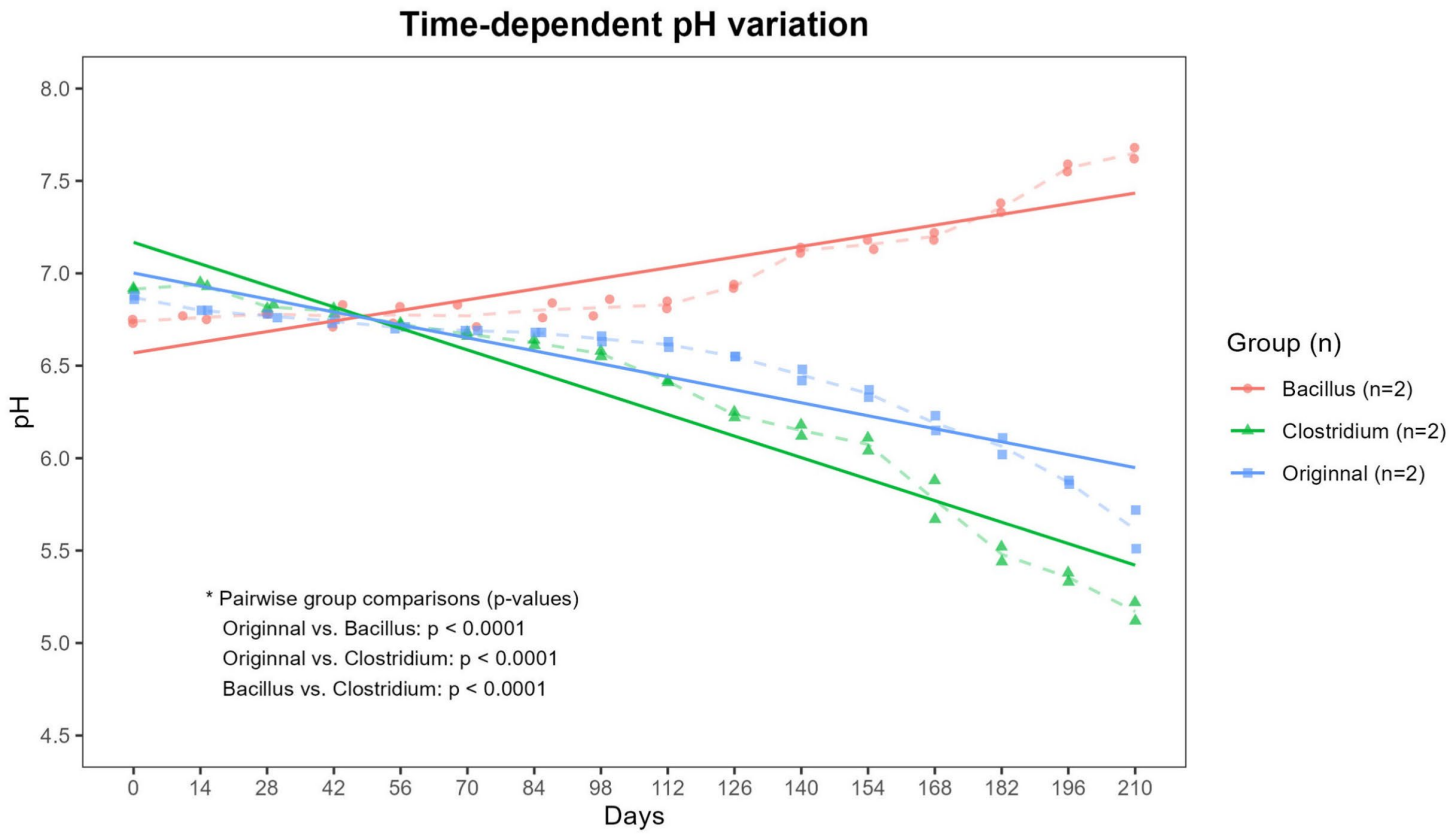
Time-dependent variation in soil pH. Changes in pH were monitored in soils treated with *Bacillus subtilis* (Bacillus), *Clostridium perfringens* (Clostridium), and the native microbial community. Soil pH was measured at regular intervals over a 210-day period. Dashed lines indicate the mean values for each group at each time point, while solid lines represent the trend lines for each group. The Original and Clostridium groups exhibited a decreasing trend in pH over time, whereas the Bacillus group showed an increasing trend. These trends of pH change were significantly different among groups (*p* < 0.001).

To assess the impact of microbial inoculation on soil chemical properties, pH measurements were performed every 2 weeks over the 210-day incubation period. As shown in Figure 1, a clear divergence in soil pH was observed among the three treatment soils (*p* < 0.0001). In the Bacillus-inoculated soil, the soil pH gradually increased from an initial value of approximately 6.75 to 7.6 by day 210, indicating a shift toward slightly alkaline over time. In contrast, the Clostridium-inoculated soil exhibited a marked acidification trend, with pH decreasing steadily from about 6.9 at the start to below 5.5 by the end of the experiment. The original group, which contained the native microbial community, showed a moderate decline in pH, reaching approximately 6.0 at day 210. These results suggest that the metabolic activities of the inoculated bacterial strains had a profound influence on the surrounding soil environment, potentially altering microbial degradation pathways through pH modulation.

### 3.2 Time-dependent degradation of mulching films weight

**Figure 2:**
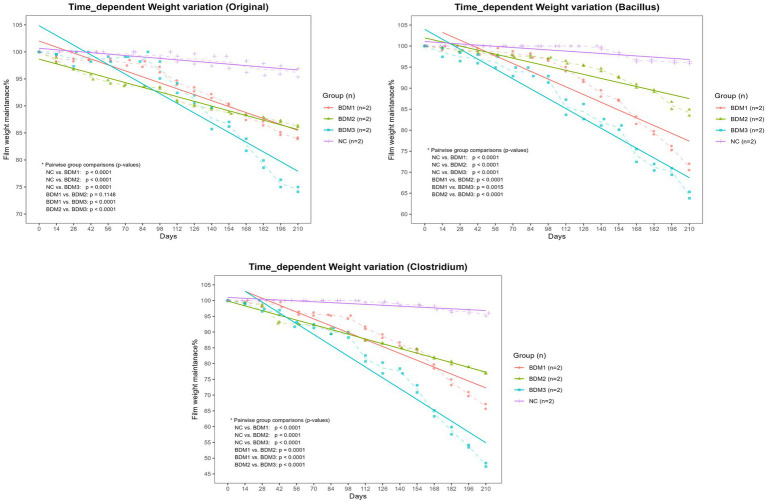
Time-dependent biodegradation of films in different soil conditions. The percentage of film weight remaining was monitored for BDM1, BDM2, BDM3, and the negative control (NC) films incubated in soils containing the native community (Original), *Bacillus subtilis* (Bacillus), or *Clostridium perfringens* (Clostridium). Dashed lines indicate the mean values for each group at each time point, while solid lines represent the trend lines for each group. BDM1, BDM2, and BDM3 exhibited significantly greater weight loss rates compared to the NC across all soil types (*p* < 0.01). Among the biodegradable films, BDM3 demonstrated a significantly higher weight loss rate than the others (*p* < 0.01).

**Figure 3:**
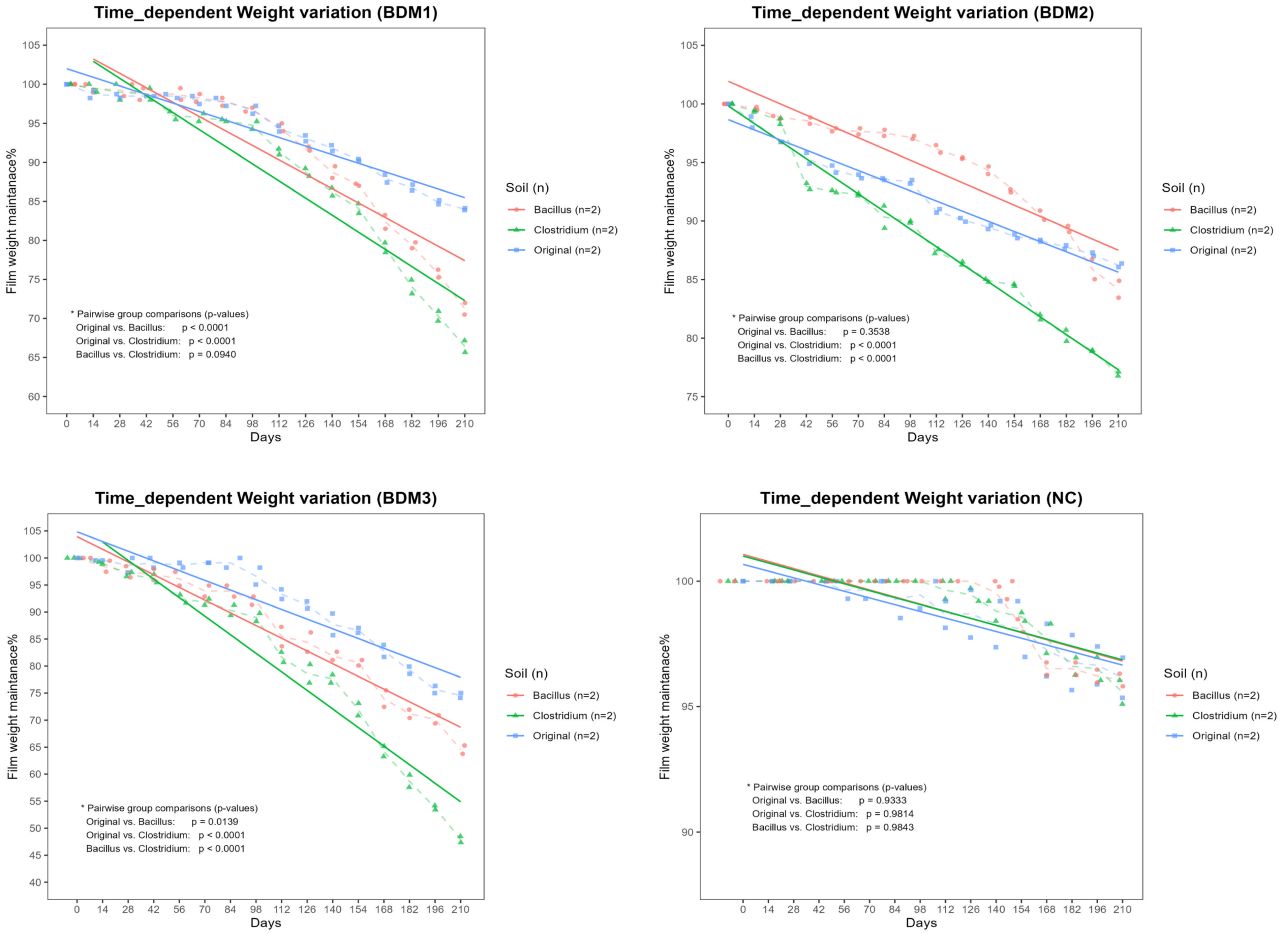
Effect of soil condition on time-dependent biodegradation of different film types. The percentage of film weight remaining was monitored for BDM1, BDM2, BDM3, and the negative control (NC) films incubated in soils containing the original microbial community (Original), *Bacillus subtilis* (Bacillus), or *Clostridium perfringens* (Clostridium). Dashed lines indicate the mean values for each group at each time point, while solid lines represent the trend lines for each group. The Clostridium-inoculated soil exhibited significantly greater biodegradation than the other soil types for all biodegradable film types except BDM1 (*p* < 0.0001), where there was no significant difference in biodegradation between Clostridium-inoculated and Bacillus-inoculated soils.

As a result of comparing the degradation levels of mulching films by soil conditions, it was found that in all soil conditions, During the 210-day period, the BDMs showed a significantly greater reduction in weight compared to the NC (*p* < 0.0001, Figure 2). Among the BDMs, BDM3 exhibited the highest weight loss (*p* < 0.01, Figure 2). When observing the degradability by soil condition in each BDM, for BDM1, the soils inoculated with Clostridium and Bacillus showed higher biodegradability than the original soil (*p* < 0.0001). For BDM2, the Clostridium-inoculated soil exhibited the highest degradability (*p* < 0.0001), while there was no significant difference in biodegradability between the Bacillus-inoculated soil and the original soil. For BDM3, the Clostridium-inoculated soil also had the highest degradability (*p* < 0.0001), followed by the Bacillus-inoculated soil and then the original soil (*p* < 0.05). Overall, the Clostridium-inoculated soil exhibited the most superior degradability of the films among the soil conditions (Figure 3).

### 3.3 Time-dependent changes in bacterial populations (CFU/g) in soil

**Figure 4:**
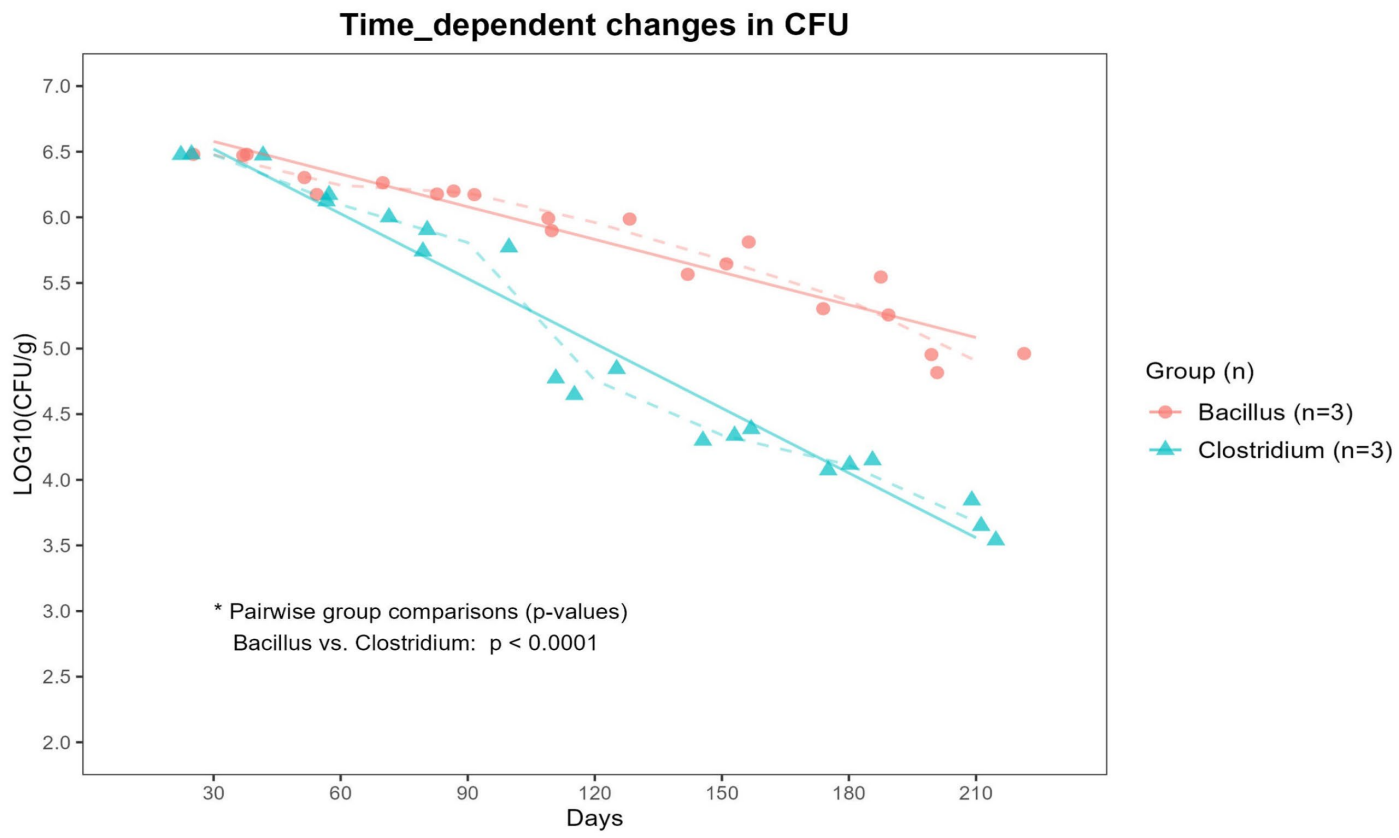
Time-dependent changes in CFU counts of *Bacillus subtilis* and *Clostridium perfringens* over 7 months. Soil samples inoculated with either *Bacillus subtilis* (Bacillus) or *Clostridium perfringens* (Clostridium) were sampled monthly to determine viable cell counts, expressed as log10 CFU/g of soil. Dashed lines indicate the mean values for each group at each time point, while solid lines represent the trend lines for each group. While both strains exhibited a gradual decline in CFU over time, *Clostridium perfringens* showed a significantly sharper decrease compared to *Bacillus subtilis* (*p* < 0.0001).

To assess the persistence of the inoculated bacterial strains over time, viable CFUs of *B. subtilis* and *C. perfringens* were quantified every 30 days during the 210-day period. As shown in Figure 4, both bacterial strains exhibited a gradual decline in population over time. However, the reduction rate varied significantly between the two groups and *C. perfringens* was found to decrease more markedly than *B. subtilis* (Figure 4).

### 3.4 SEM-based morphological evaluation and quantitative surface damage analysis

**Figure 5:**
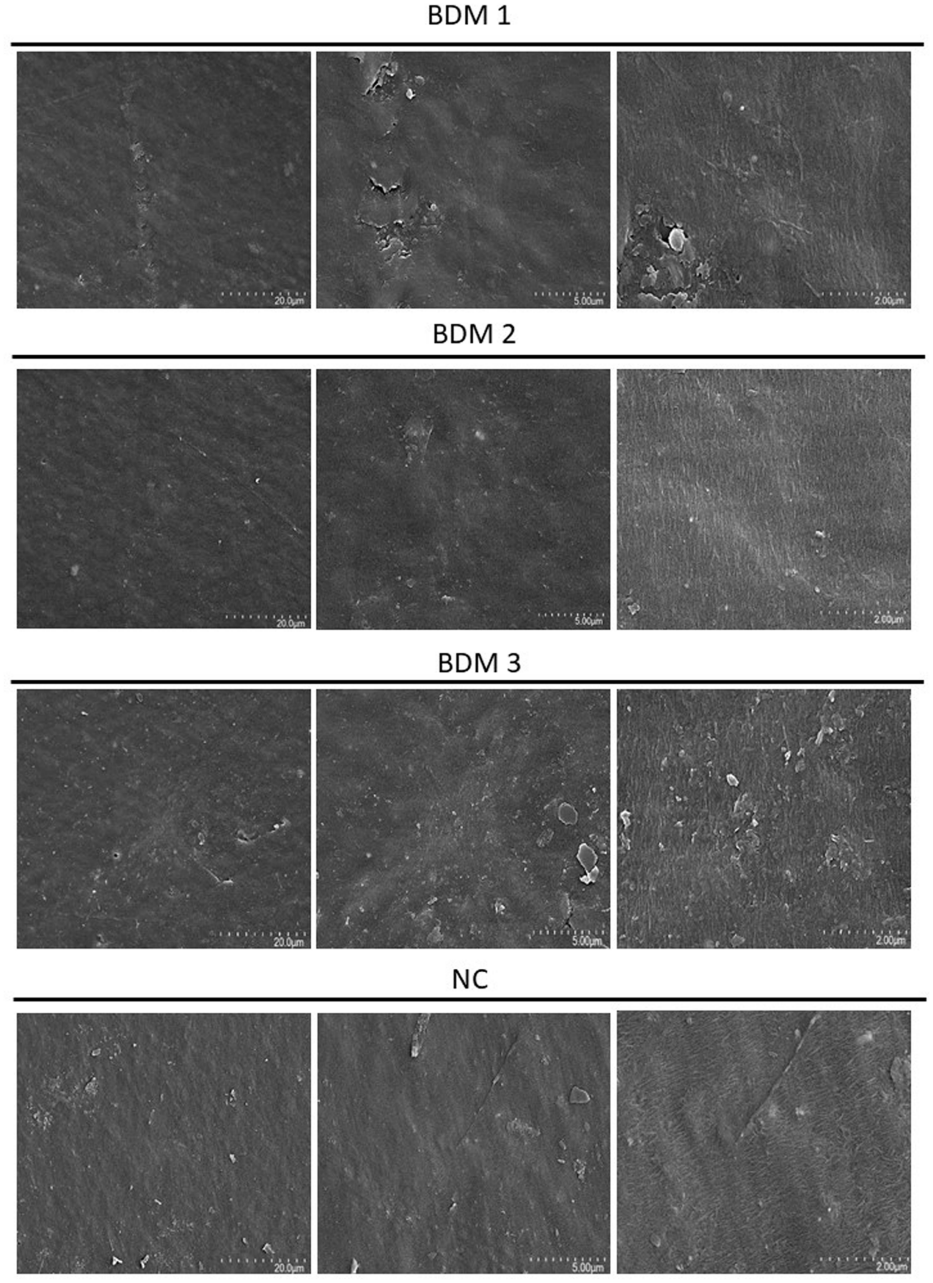
Surface morphology of BDM1, BDM2, BDM3, and NC film types observed using scanning electron microscopy (SEM) after 210 days of incubation in soil treated with the *Clostridium perfringens*. Images were captured at three magnifications: 3,000 × (left), 10,000 × (middle), and 30,000 × (right).

**Figure 6:**
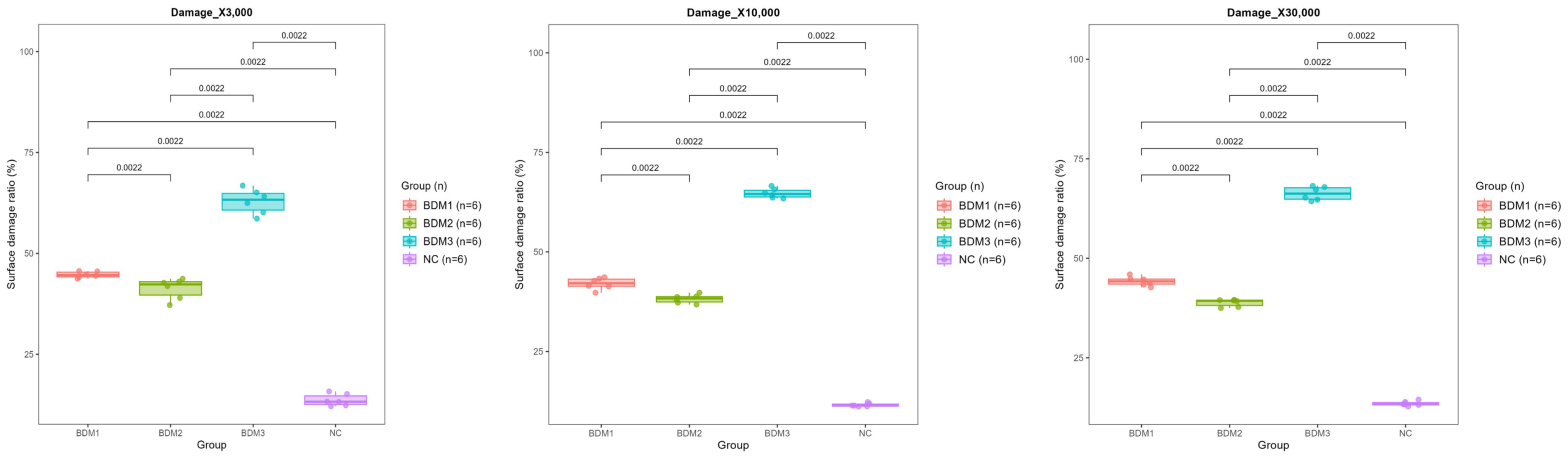
Surface damage ratios (%) of each mulching film quantified after 210 days of incubation in Clostridium-inoculated soil. Damage quantification was performed by analyzing SEM images taken at three magnifications (×3,000, ×10,000, and ×30,000). For each film, ImageJ software was used to calculate the proportion of disrupted surface area in three randomly selected regions. Pairwise statistical analysis revealed that all groups displayed significant differences from each other at all magnifications. Among the film types, BDM3 exhibited the highest surface damage ratios at every field.

SEM was conducted to assess the structural degradation of biodegradable films under microbial influence. The Clostridium–inoculated group exhibited the most significant film weight loss across all soil types. Therefore, this group was selected for further surface morphological analysis. SEM imaging at three magnifications (×3,000, ×10,000, and ×30,000) revealed progressively more detailed damage characteristics across BDM1, BDM2, BDM3, and NC films. The biodegradable films displayed extensive surface erosion, including holes, cracks, and rough textures, whereas the NC film maintained relatively smooth surfaces with minimal visible degradation (Figures 5, 6). Quantification of surface damage was performed using ImageJ software across all three magnifications. Surface-damaged regions were measured and calculated as a percentage of the total film area. BDM3 exhibited the highest average surface damage ratio across magnifications, followed by BDM1 and BDM2, all significantly higher than NC (*p* < 0.01). These trends were consistently observed across all magnifications, confirming the robustness of the observed degradation patterns. These findings demonstrate that BDM3 is the most susceptible to microbial surface degradation among the film types.

## 4 Discussion

### 4.1 Selection of inoculant bacteria

The Bacillus genus is ubiquitous in soil, where it contributes to nutrient cycling, plant growth promotion, and protection against pathogens and environmental stresses (Saxena et al., 2020). Among Bacillus species, *B. subtilis* stands out for its well-characterized physiology and frequent use as a model organism in soil microbiology research, owing to its ease of cultivation and genetic tractability (Earl et al., 2008). Accordingly, *B. subtilis* was selected in this study as a representative facultative anaerobe.

The Clostridium genus is also widely distributed in natural environments, particularly in soils (Choi et al., 2004; Chalmers et al., 2008). Among its members, *C. perfringens* is commonly detected in agricultural soils, animal manure, and compost (Choi et al., 2004; Mehdizadeh Gohari et al., 2021). Its notable ability to form resilient endospores allows it to survive prolonged exposure to adverse conditions such as heat, desiccation, and oxygen (Choi et al., 2004; Chalmers et al., 2008). Based on these ecological and physiological traits, *C. perfringens* was chosen as the representative anaerobe for this study.

### 4.2 Relationship between biodegradable film decomposition and soil pH

Soil pH is a critical modulator of microbial enzymatic activity and polymer degradation efficiency (Lucas et al., 2008). In this study, *B. subtilis* inoculation led to a slight increase in soil pH, consistent with its known production of ammonia and proteolytic enzymes (Setlow, 2016; Baskaran and Sathiavelu, 2022). Nonetheless, the pH remained within the neutral range (approximately 6.75 to 7.6) over the entire period. In contrast, *C. perfringens* significantly acidified the soil, likely due to anaerobic fermentation and short-chain fatty acid (SCFA) production (Khoruts et al., 2021; Schnizlein and Young, 2022). These divergent pH shifts strongly correlated with degradation outcomes: the Clostridium-induced acidic conditions accelerated film weight loss regardless of the BDM types, while Bacillus-induced near-neutral conditions led to more moderate degradation (Figure 3).

Biodegradation by microorganisms proceeds most efficiently under neutral pH conditions, as most microbial enzymes exhibit optimal activity at neutral pH and their function is generally inhibited in acidic environments (Lucas et al., 2008; Setlow, 2016; Bandopadhyay et al., 2023). However, in terms of chemical degradation, acidic conditions can be advantageous (Lucas et al., 2008; Haider et al., 2019). The components of the BDMs (PBAT, PLA, TPS, and CaCO₃) undergo chemical degradation that is mildly to strongly accelerated under acidic conditions (Kale et al., 2007; Lucas et al., 2008; Haider et al., 2019). Especially, acid significantly promotes the hydrolysis of TPS and rapidly dissolves CaCO₃ (Kale et al., 2007; Wang et al., 2021; Bandopadhyay et al., 2023). In this study, although the viable cell counts of *C. perfringens* in the Clostridium-inoculated soil declined sharply after day 90, The soil pH steadily decreased until day 210, exhibiting a tendency to drop more sharply after 90 days. This is likely due to the accumulation of organic acids produced by anaerobic fermentative bacteria during the period of active microbial metabolism. These organic acids remained in the soil, persistently acidifying the environment even after the microbial population had decreased (Kale et al., 2007; Bandopadhyay et al., 2023). In the Clostridium-inoculated soils, the weights of BDM1 and BDM3, both containing CaCO₃, exhibited a more pronounced decrease after day 90, similar to the pH change pattern (Figure 2; Clostridium). Considering the low viable cell counts during the period after day 90, this marked weight loss is thought to be due primarily to chemical degradation under acidic conditions rather than to biodegradation by microorganisms. The acidic condition is thought to have rapidly dissolved the CaCO₃ present in BDM1 and BDM3, which in turn disrupted the film structure and accelerated degradation. These results suggest that manipulating soil conditions – for instance, using microbes or amendments to adjust pH – could be a viable strategy to steer biodegradation.

### 4.3 Comparison of weight loss depending on the biodegradable film type

In this study, *B. subtilis* maintained a higher population than *C. perfringens* throughout the 210-day period. The soil surface in which the experiment was conducted is likely to be under aerobic conditions due to efficient air exchange and this persistence is likely attributable to the facultative anaerobic characteristics of *B. subtilis* (Setlow, 2016), which permit survival under variable oxygen conditions, as opposed to the strictly anaerobic *C. perfringens* (Choi et al., 2004). Furthermore, while the degradative capacity of Bacillus for PBAT, PLA, and TPS has been well established, there is limited knowledge regarding the ability of Clostridium species to degrade these materials, and their efficiency is generally presumed not to be superior to that of Bacillus (Lucas et al., 2008; Bandopadhyay et al., 2023; Fernandes, 2023). Despite the low population of *C. perfringens*, all BDMs exhibited the greatest weight loss in the Clostridium-inoculated soil, suggesting that the synergistic effects of direct microbial degradation and acid-induced chemical degradation contributed to this pronounced outcome (Figure 3).

When comparing the weight loss rates of the BDM types, BDM3 exhibited the highest weight loss across all soil conditions (Figure 2), thus it was regarded as the most appropriate BDM when both chemical and microbial degradation were considered. Several studies have reported that monolayer (homogeneous) films provide a greater surface area for microbial contact and present fewer barriers, resulting in a higher degradation rate compared to compound (layered) films (Bandopadhyay et al., 2023; Fernandes, 2023). BDM3, as a monolayer and homogeneous structure, allows microbes and enzymes to directly access the entire film matrix, enabling rapid degradation not only at the surface but also within the interior of the film. Additionally, the simple blend configuration of BDM3 means that CaCO₃ can quickly react to acid attacks (microbial metabolites and organic acids) leading to rapid disintegration of the overall structure. This increases the likelihood that PLA and PBAT are simultaneously exposed to degradation environments (e.g., enzymatic activity and pH changes). In contrast, BDM1 and 2 were designed as a three-layer structure, with structural barriers at each interfacial layer (Crétois et al., 2017). In particular, the middle layer of BDM1, consisting exclusively of 100% PBAT without CaCO₃, exhibits greater resistance to external acidification. Such a multilayered and complex structure impedes the penetration and diffusion of microbes or enzymes, thereby hindering uniform and efficient degradation throughout the film (Messin et al., 2017; Bandopadhyay et al., 2023; Zhang et al., 2024). When comparing the weight loss of the three-layer BDMs, BDM1 exhibited greater degradation than BDM2 in the Bacillus-inoculated soil, where the pH remained nearly neutral (*p* < 0.0001, Figure 2; Bacillus). As acidic conditions were not expected to affect film degradation in this soil, the weight loss is likely attributable primarily to microbial biodegradation rather than chemical degradation. This finding appears to be attributable to structural differences arising from the compositional components of each film. BDM1, due to the incorporation of CaCO_3_, form stable and persistent microvoids throughout the films from the manufacturing stage. This porous structure is consistently maintained, allowing microbial enzymes to actively penetrate the film matrix and thus promoting efficient biodegradation (Messin et al., 2017; Bandopadhyay et al., 2023; Zhang et al., 2024). In contrast, while the TPS component in BDM2 is rapidly decomposed in the early stages, creating temporary pores, these pores tend to shrink, collapse, or become blocked over time due to factors such as soil pressure and moisture. As a result, although BDM2 initially exhibits rapid degradation, its weight loss slows considerably in the long term. Ultimately, these structural differences arising from compositional variations are presumed to be responsible for the higher weight loss observed in BDM1 compared to BDM2 (Messin et al., 2017; Bandopadhyay et al., 2023; Zhang et al., 2024).

The inclusion of both mono-layer and multi-layer BDMs in our study thus provides practical insight: real-world biodegradable mulches often have multi-layer designs for strength and longevity, and our results demonstrate that while such designs are more degradation-resistant, they do eventually biodegrade under enriched microbial activity. Although single-layer films are clearly advantageous in terms of biodegradation efficiency, practical field applications require consideration of multiple factors such as mechanical durability, functional performance, and controllability (Bandopadhyay et al., 2023). It has been suggested that multilayer structures may be essential to ensure sustained mulching function and soil stability in real agricultural settings (Bandopadhyay et al., 2023).

### 4.4 Applications and environmental risks of microbial inoculants

In the application of BDMs, complete degradation is essential for environmental sustainability. The degradation rate can vary significantly depending on climate and other environmental conditions, and even biodegradable films may not fully disappear, with microplastics or residual fragments remaining in the soil (Abbate et al., 2023). These residues, including microplastics, can have both positive and negative effects on soil organic matter, microbial activity, enzyme functions, and physical properties such as porosity and water retention. Especially when accumulated over the long term, residues may alter soil structure and nutrient transport (Abbate et al., 2023). Recent findings suggest that specific microbes such as *Pseudomonas putida* can actively colonize and degrade biodegradable mulch films in agricultural soil (Fontanazza et al., 2021). Therefore, introducing bacteria that are advantageous for BDM degradation as soil inoculants can help enhance the degradation efficiency of BDMs.

In this study, two bacterial species (*B. subtilis* and *C. perfringens*) commonly found in soil were used. Among these bacteria, the soil inoculated with *Clostridium perfringens* exhibited the highest rates of BDM degradation, suggesting that this bacterium could serve as an effective microbial inoculant for BDM breakdown. However, its practical application must be carefully evaluated considering potential ecological and public health risks.

*B. subtilis* may very rarely cause opportunistic infections, its pathogenic risk is extremely low in industrial, agricultural, and environmental contexts, and it is not considered a major pathogen (Gu et al., 2019; Lee et al., 2019). In contrast, pathogenic *C. perfringens* strains that possess toxin genes can cause diseases such as food poisoning and enteritis in humans and animals (Songer, 1996), raising concerns for food safety and public health if such strains proliferate or persist in agricultural soils. Additionally, improper use of strains harboring antibiotic resistance genes may promote the spread of resistance in the environment (Fletcher and medicine, 2015), making it essential to select non-pathogenic and antibiotic-sensitive strains. Most crops achieve optimal growth and nutrient uptake at soil pH 6.0–7.0, while excessively low or high pH can lead to reduced mineral absorption and growth impairment (O’Kennedy, 2022). Therefore, when applying *C. perfringens,* excessive soil acidification may negatively impact crop productivity, so maintaining mildly acidic conditions (pH 6.0–6.5) through proper soil pH monitoring is recommended. Although this study used artificial soils inoculated with either *C. perfringens* or *B. subtilis* individually, future research should examine whether co-inoculation of these bacteria might further promote BDM degradation while helping to maintain mildly acidic soil conditions.

## 5 Conclusion

This study compared the effects of two representative soil bacteria, *B. subtilis* and *C. perfringens*, as inoculants on the degradation efficiency of various biodegradable mulching films and their impacts on soil environment. The results demonstrated that soil inoculation with *C. perfringens* led to the highest rates of film degradation and pronounced surface damage, primarily due to strong acidification from accumulated organic acids produced by microbial metabolism. Especially in CaCO₃-containing films (BDM1 and BDM3), structural collapse following acidification was evident, indicating that acid-induced chemical degradation plays a major role even when oxidative microbial activity is limited. In contrast, *B. subtilis* maintained relatively robust viability in soil, promoted gradual film degradation, and kept soil pH within a neutral to slightly alkaline range, minimizing negative effects on plant growth. These findings suggest that soil microbial composition and pH regulation are critical for effective biodegradation of mulching films and for maintaining soil and crop health. Although *C. perfringens* application can enhance film degradation, it may also lead to excessive soil acidification, impaired plant growth, and environmental spread of pathogenic strains. Therefore, industrial application requires rigorous safety validation, continuous soil pH monitoring, and the selection of non-pathogenic strains.

## Data Availability

The raw data supporting the conclusions of this article will be made available by the authors, without undue reservation.
